# Supplementary material for: Remediating Potentially Toxic Metal and Organic Co-Contamination of Soil by Combining In Situ Solidification/Stabilization and Chemical Oxidation: Efficacy, Mechanism, and Evaluation
Source: Int J Environ Res Public Health. 2018 Nov 20;15(11):2595. doi: 10.3390/ijerph15112595 (PMC6266821; doi:10.3390/ijerph15112595)
Supplement: Supplementary file 1 [file ijerph-15-02595-s001.pdf]

Table S1. Basic conditions of select contaminated sites in China [1].

Serial Number	Historical Use	Plan for Future Use	Restoration Medium	Principle Pollutants
S1	Chemical plant	Residential land	Soil	Trichlorobenzene, hexachlorobenzene, and As
S2	Chemical plant	Industrial land	Soil	Aniline, TPH, Cd, and As
S3	Chemical plant	Residential land	Soil	Benzene, TPH, benzopyrene, hexachlorocyclohexane, As, and Cr
S4	Chemical plant	Municipal traffic	Soil	Tetrabutyl tin, DDTs, PAHs, Pb, and Cr
S5	Electronic waste plant	Industrial land	Soil	Aniline, Tetrabromobisphenol A, and Cd
S6	Electronic waste plant	Industrial land	Soil	PCBs, PAHs, and Cd

DDT: dichlorodiphenyltrichloroethane; PAH: polycyclic aromatic hydrocarbon; PCB: polychlorinated biphenyl; TPH: total petroleum hydrocarbons.

Table S2. Physical and chemical properties of the tested soil.

Physicochemical Property	Soil Organic Matter (g/kg)	Cation Exchange Capacity (cmol/kg)	Electrical Conductivity (μS/cm)	Water Content (%)	Porosity (%)	pH
Value	18.91	6.19	0.33	7.63	50.50	7.49

Table S3. Reaction scenarios tested, corresponding names, and amendments doses (amendment weight/dry soil weight).

Reaction Scenario	Reactor Name	PS (mol)	ISS (%)	$Ca(OH)_2$ (%)
Unactivated PS	PS	1.08	0	0
$Ca(OH)_2$-activated PS	$Ca(OH)_2$ + PS	1.08	30	30
Heat-activated PS	Heat + PS	1.08	30	0
QL	QL	0	30	0
PC	PC	0	30	0
BFS	BFS	0	30	0
FA	FA	0	30	0
QL-activated PS	QL + PS	1.08	30	0
PC-activated PS	PC + PS	1.08	30	0
BFS-activated PS	BFS + PS	1.08	30	0
FA-activated PS	FA + PS	1.08	30	0
QL	5% QL	0	5	0
QL	10% QL	0	10	0
QL	20% QL	0	20	0
QL	30% QL	0	30	0
QL	50% QL	0	50	0
QL-activated PS	5% QL + PS	1.08	5	0
QL-activated PS	10% QL + PS	1.08	10	0
QL-activated PS	20% QL + PS	1.08	20	0
QL-activated PS	30% QL + PS	1.08	30	0
QL-activated PS	50% QL + PS	1.08	50	0
PC	5% PC	0	5	0
PC	10% PC	0	10	0
PC	20% PC	0	20	0
PC	30% PC	0	30	0
PC	50% PC	0	50	0
PC-activated PS	5% PC + PS	1.08	5	0

PC-activated PS	10% PC + PS	1.08	10	0
PC-activated PS	20% PC + PS	1.08	20	0
PC-activated PS	30% PC + PS	1.08	30	0
PC-activated PS	50% PC + PS	1.08	50	0

BFS: blast-furnace slag; FA: fly ash; ISS: in situ solidification/stabilization; PC: Portland cement; PS: persulfate; QL: quick lime.

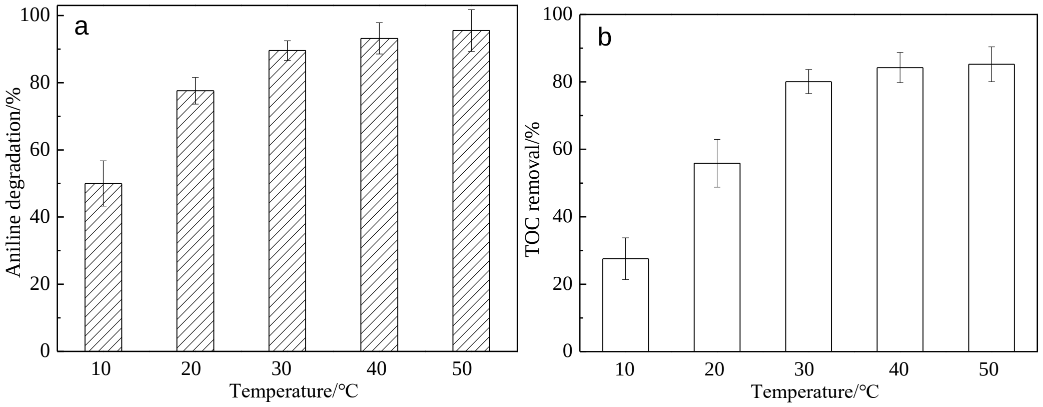

Figure S1. Effect of temperature on (**a**) aniline degradation (reaction time: 10 min) and (**b**) total organic carbon (TOC) removal in soil (reaction time: 210 min).

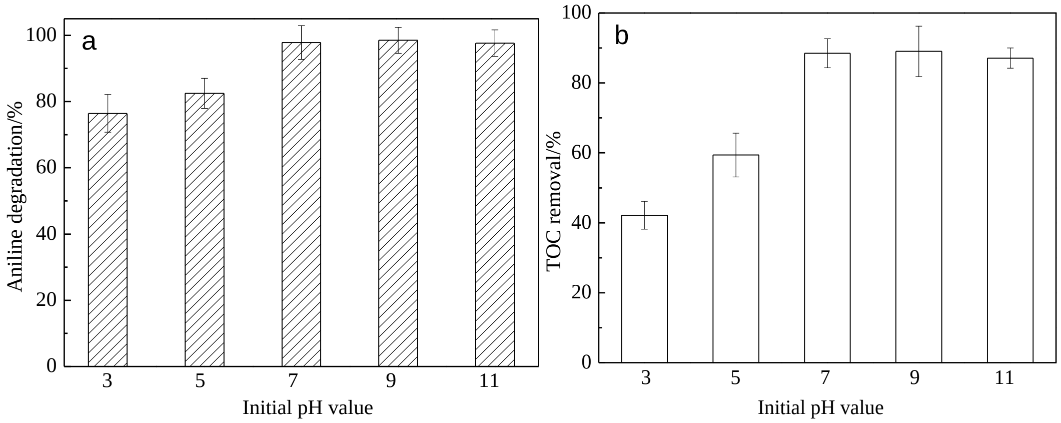

Figure S2. Effect of pH on (**a**) aniline degradation (reaction time: 10 min) and (**b**) total organic carbon (TOC) removal in soil (reaction time: 210 min).

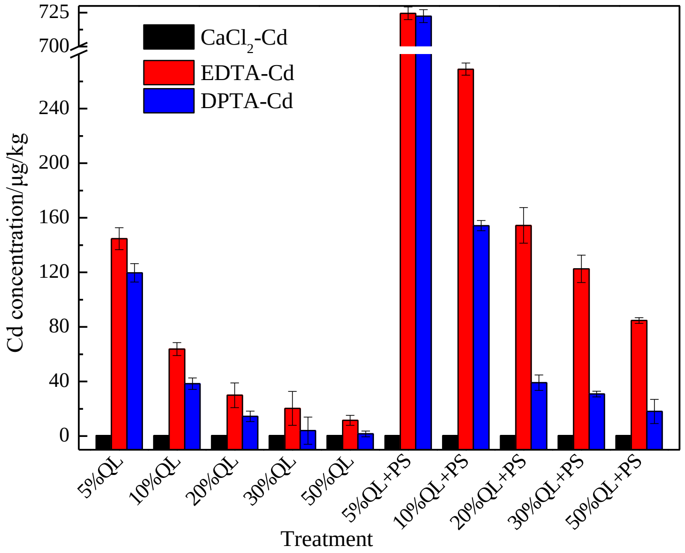

Figure S3. Effect of different dosages of quick lime on the bioavailable Cd content in co-contaminated soil. Quick lime (QL), Persulfate (PS).

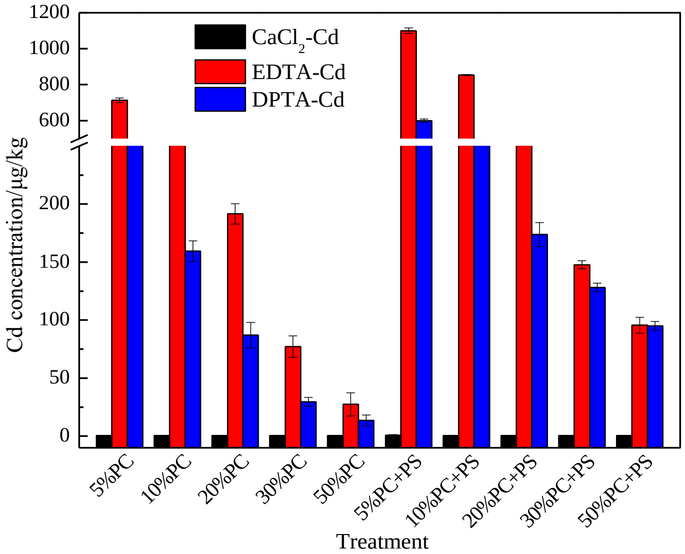

Figure S4. Effect of different dosages of Portland cement on the bioavailable Cd content in co-contaminated soil. Portland cement (PC), Persulfate (PS).

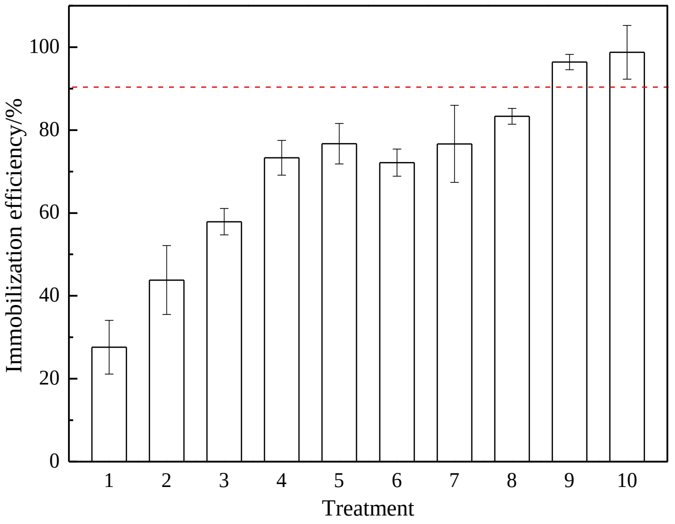

1-5%QL；2-10%QL；3-20%QL；4-30%QL；5-50%QL；6-5%QL+PS
7-10%QL+PS；8-20%QL+PS；9-30%QL+PS；10-50%QL+PS

Figure S5. Effect of different dosages of quick lime on the immobilization efficiency of aniline in co-contaminated soil. See Table S3 for the treatment definitions. Quick lime (QL), Persulfate (PS).

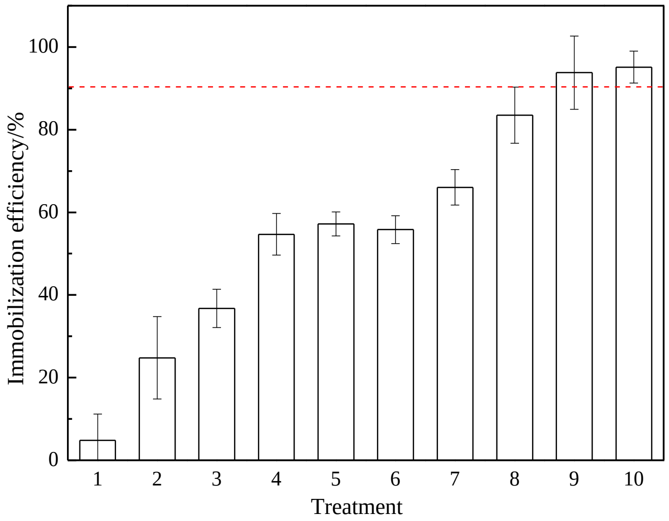

1-5%PC；2-10%PC；3-20%PC；4-30%PC；5-50%PC；6-5%PC+PS；
7-10%PC+PS；8-20%PC+PS；9-30%PC+PS；10-50%PC+PS

Figure S6. Effect of different dosages of Portland cement on the immobilization efficiency of aniline in co-contaminated soil. See Table S3 for the treatment definitions. Portland cement (PC), Persulfate (PS).

Reference

1 Ma, Y.; Wang, D.; Xu, Z.; Dong, B.B.; Chen, J.Q.; Xie, Y.F.; Li, F.S.; Du, X.M. Current situation, problems and countermeasures of industrial contaminated sites remediation in Beijing. *Environ. Eng.* **2017**, *35*, 120–124.
